# Comparative examination of various PCR-based methods for *DNMT3A* and *IDH1/2* mutations identification in acute myeloid leukemia

**DOI:** 10.1186/1756-9966-33-44

**Published:** 2014-05-21

**Authors:** Rimma Berenstein, Igor Wolfgang Blau, Asiye Kar, Ruhiye Cay, Annette Sindram, Claudia Seide, Olga Blau

**Affiliations:** 1 Department of Hematology, Oncology and Tumourimmunology, Charité Universitätsmedizin Berlin, Hindenburgdamm 30, 12200 Berlin, Germany; 2 Department of Biotechnology, Beuth University of Applied Sciences, Luxemburger Strasse 10, 13353 Berlin, Germany; 3 Labor Berlin, Department of Hematology and Oncology, Charité Universitätsmedizin Berlin, Sylterstrasse 2, 13353 Berlin, Germany

**Keywords:** AML, *DNMT3A*, *IDH1/2* mutations, DNA sequencing, HRM analysis, ARMS PCR, Endonuclease restriction

## Abstract

**Background:**

Mutations in epigenetic modifiers were reported in patients with acute myeloid leukaemia (AML) including mutations in DNA methyltransferase 3A gene (*DNMT3A)* in 20%-30% patients and mutations in isocitrate dehydrogenase 1/2 gene (*IDH1/2*) in 5%-15% patients. Novel studies have shown that mutations in *DNMT3A* and *IDH1/2* influence prognosis, indicating an increasing need to detect these mutations during routine laboratory analysis. DNA sequencing for the identification of these mutations is time-consuming and cost-intensive. This study aimed to establish rapid screening tests to identify mutations in *DNMT3A* and *IDH1/2* that could be applied in routine laboratory procedures and that could influence initial patient management.

**Methods:**

In this study we developed an endonuclease restriction method to identify the most common *DNMT3A* mutation (R882H) and an amplification-refractory mutation system (ARMS) to analyse *IDH2* R140Q mutations. Furthermore, we compared these methods with HRM analysis and evaluated the latter for the detection of *IDH1* mutations.

**Results:**

Of 230 samples from patients with AML 30 (13%) samples had *DNMT3A* mutations, 16 (7%) samples had *IDH2* R140Q mutations and 36 (16%) samples had *IDH1* mutations. Sensitivity assays performed using serial dilutions of mutated DNA showed that ARMS analysis had a sensitivity of 4.5%, endonuclease restriction had a sensitivity of 0.05% and HRM analysis had a sensitivity of 5.9%–7.8% for detecting different mutations. HRM analysis was the best screening method to determine the heterogeneity of *IDH1* mutations. Furthermore, for the identification of mutations in *IDH2* and *DNMT3A,* endonuclease restriction and ARMS methods showed a perfect concordance (100%) with Sanger sequencing while HRM analysis showed a near-perfect concordance (approximately 98%).

**Conclusion:**

Our study suggested that all the developed methods were rapid, specific and easy to use and interpret. HRM analysis is the most timesaving and cost-efficient method to rapidly screen all the 3 genes at diagnosis in samples obtained from patients with AML. Endonuclease restriction and ARMS assays can be used separately or in combination with HRM analysis to obtain more reliable results. We propose that early screening of mutations in patients with AML having normal karyotype could facilitate risk stratification and improve treatment options.

## Background

Acute myeloid leukaemia (AML) is a clonal disorder characterised by the accumulation of myeloid cells and impairment of normal haematopoiesis [1]. The recent large-scale sequencing of AML genomes is now providing opportunities for patient stratification and personalised approaches to treatments that are based on an individual’s mutation profiles [1-3]. A few recurring gene mutations and overexpressed genes having prognostic relevance in AML have been identified and incorporated in the current prognostication models.

Recently, a new class of mutations affecting genes for DNA methylation and post-translational histone modification was identified in AML. These mutations frequently occur in the DNA nucleotide methyltransferase 3A gene (*DNMT3A*) [4-8] and isocitrate dehydrogenase 1/2 gene (*IDH1/2)* (isocitrat dehydrogenase 1/2) [9-13]. *DNMT3A* belongs to the mammalian methyltransferase gene family, which also includes *DNTM1*, *DNMT3B* and *DNMT3L*. Methyltransferases modify methylation patterns by enzymatically adding a methyl group to cytosine residues in CpG islands and are involved in tissue-specific gene expression [4,14]. Studies in different AML cohorts have reported the incidence of *DNMT3A* mutations in up to 22% *de novo* AML and 36% cytogenetically normal AML samples [5,6]. Nonsense, frameshift and missense mutations commonly occur in *DNMT3A*; however a point mutation at position R882 is the most frequently (40%–60%) observed mutation [7]. *In vitro* studies suggest that mutations at this position disturb the formation of heterodimers with *DNMT3L,* thereby preventing the catalytic activity of *DNMT3A*. Different studies have shown a negative impact of *DNMT3A* mutation on outcomes in patients with AML [3,15-19]. Prognostic effect is known to depend on certain biological factors as well as a combination of cytogenetics and other mutations such as those in *FLT3* and *NPM1*[3,6,8].

Somatic mutations in *IDH1/2* occur in 5–30% patients with AML and are commonly associated with nucleophosmin 1 (*NPM1)* mutations [9,10]. Both the genes play a critical role in the citric acid cycle *IDH1* in the cytoplasm and peroxisome and *IDH2* in the mitochondria. Both *IDH1* and *IDH2* promote the conversion of isocitrate to α-ketoglutarate (α-KG) that is associated with the reduction of nicotinamide adenine dinucleotide phosphate (NADP^+^) to NADPH [8,11,20]. Mutations in *IDH1* and *IDH2* are heterozygous and occur in highly conserved arginine residues (*IDH1 R132* and *IDH2 R140/R172*). Mutations at *IDH2 *R140 always result in the conversion of arginine to glutamine, whereas substitutions at *IDH1 *R132 and *IDH2 *R172 result in a wide range of amino acid replacements [12]. All point mutations in *IDH1/2* lead to a gain of function, enabling the conversion of α-KG to 2-hydroxyglutarate (2-HG) and oxidation of NADPH to NADP^+^. Furthermore, an increase in 2-HG-levels leads to the functional impairment of α-KG-dependent enzymes through competitive inhibition [13].

In contrast to the impact of *DNMT3A* mutations, the impact of *IDH1/2* mutations on prognosis is not completely understood. It appears that prognosis may depend on specific patient populations and a combination with *NPM1* mutations [21-23].

The increasing evidence of high incidence particularly in cytogenetically normal AML and prognostic pertinence of *DNMT3A* and *IDH1/2* mutations support the need to identify these mutations in routine diagnostic screening. Importantly, the presence of *DNMT3A* and *IDH1/2* mutations may confer sensitivity to novel therapeutic approaches, including demethylating agents [24,25].

The current available methods like direct sequencing are informative but time consuming and cost intensive. In this study, we validated the polymerase chain reaction (PCR)-based high resolution melt (HRM) assay for screening *DNMT3A*, *IDH1* and *IDH2* mutations in samples obtained from patients with AML at diagnosis and developed 2 rapid methods for detecting more common mutations, *DNMT3A* R882H and *IDH2* R140Q. We evaluated the utility of endonuclease restriction-based detection method to identify mutations in *DNMT3A* and designed an amplification-refractory mutation system (ARMS) to detect mutations in *IDH2*. In addition we compared both the systems with the HRM assay for all the studied mutations.

## Methods

### Patient characteristics

Bone marrow (BM) samples from 230 patients with newly diagnosed AML were included in the study. All patients were treated at the University Clinic Charité, Campus Benjamin Franklin, from May 2000 to July 2013. Patient’s characteristics are summarised in the Additional file 1: Table S1. The male/female ratio of the study population was 116/114, and the median age was 57 years (range, 16–94 years). Diagnoses were established according to the WHO criteria [26]. Written informed consent was obtained from all patients in accordance with the Declaration of Helsinki and the ethical guidelines of the Charite University School of Medicine, which approved this study.

### DNA extraction

Mononuclear cells from BM aspirates were isolated using Ficoll density gradient centrifugation as described [27]. DNA was extracted using Allprep DNA/RNA mini kit (Qiagen) as per the manufacturer’s instructions.

### ARMS analysis of *IDH2*-R140Q mutations

All primers were designed using Primer 3 Software (Additional file 2: Table S2). ARMS analysis was performed using 2 control primers flanking exon 23 and 2 allele-specific primers *IDH2*-RI and *IDH2*-FI that are complementary to the wild-type (wt) and mutated alleles, respectively. To enhance specificity, both the primers had an additional medium mismatch at the preliminary base. The PCR mixture and reaction conditions are specified in the Additional file 3: PCR reaction mixtures and conditions. The generated PCR products were analysed on a 1.5% agarose gel.

### Endonuclease restriction analysis of *DNMT3A*-R882H mutations

PCR amplification for endonuclease restriction analysis was conducted using primers *DNMT3A*-ResF/R (Additional file 2: Table S2). PCR reaction mixture was prepared as that described for ARMS assay. The reaction conditions are specified in the Additional file 3. In all, 10 μl of the PCR product was directly applied for endonuclease treatment with 1 μl Fnu4HI and 5 μl of CutSmart Buffer (New England Biolabs). After incubation at 37°C for 15 min products were analysed on a 1.5% agarose gel containing 10% ethidium bromide (voltage 150 V).

### HRM assay

The reaction mixture and HRM conditions are specified in the Additional file 3. The analysis was performed in a Rotor Gene 6000 Real-Time PCR Cycler (Qiagen). Samples, including a control sample for each mutation and wt allele, were analysed in duplicates. For *DNMT3A* and *IDH2*, the wt allele was used for normalisation, while for *IDH1* R132C mutation control was used as the baseline. Normalisation regions for the optimal detection of *DNMT3A* were 82°C-83°C (leading range) and 87°C-88°C (trailing range), for the optimal detection of *IDH1* were 73°C-74°C (leading range) and 82°C-83°C (trailing range) and for the optimal detection of *IDH2* were 77°C-78°C (leading range) and 87°C-88°C (trailing range). Confidence threshold was set to 70% for all the genes.

### DNA sequencing

All the primers used for sequencing are listed in the Additional file 2: Table S2. All PCR reaction conditions are specified in the Additional file 3. The obtained products were purified using the PCR Purification Kit (Qiagen), as described in the manual. Sequencing reaction was performed using Big Dye Terminator v3.1 Cycle Sequencing Kit (Applied Biosystems). The sequencing products were purified using DyeEx 2.0 Spin Kit (Qiagen) according to the manufacturer’s instructions. The purified products were diluted with 18 μl HiDi-Formamid (Applied Biosystems), incubated at 95°C for 3 min and chilled on ice for 3 min. Sequencing was performed using ABI310 Genetic Analyser (Applied Biosystems), and data were collected using ABI Prism 310 Data Collection Software.

## Results and discussion

All the positive and negative controls used in this study were selected by Sanger sequencing of patients’ samples. The results obtained using endonuclease restriction, ARMS and HRM were verified with those obtained using Sanger sequencing to determine the specificity of the assays. Sensitivity was measured as the minimal percentage of mutated allele in a sample detected by the assay. The initial portion of mutation was determined using Sanger sequencing.

### *DNMT3A* mutation analysis

Endonuclease restriction analysis identified *DNMT3A* R882H G>A mutations in 28 out of 230 patients with AML (12.2%) and HRM analysis identified 2 additional R882X G>C mutations (0.9%), which are consistent with the frequency published by Lin et al. [28]. The age of the patients ranged from 24 to 87 years (median, 58 years). Among these patients, 53% had a normal karyotype. None of the patients in the prognostic favourable group had *DNMT3A* mutations. Of 30 patients, 16 had *FLT3* mutations.

**Figure 1:**
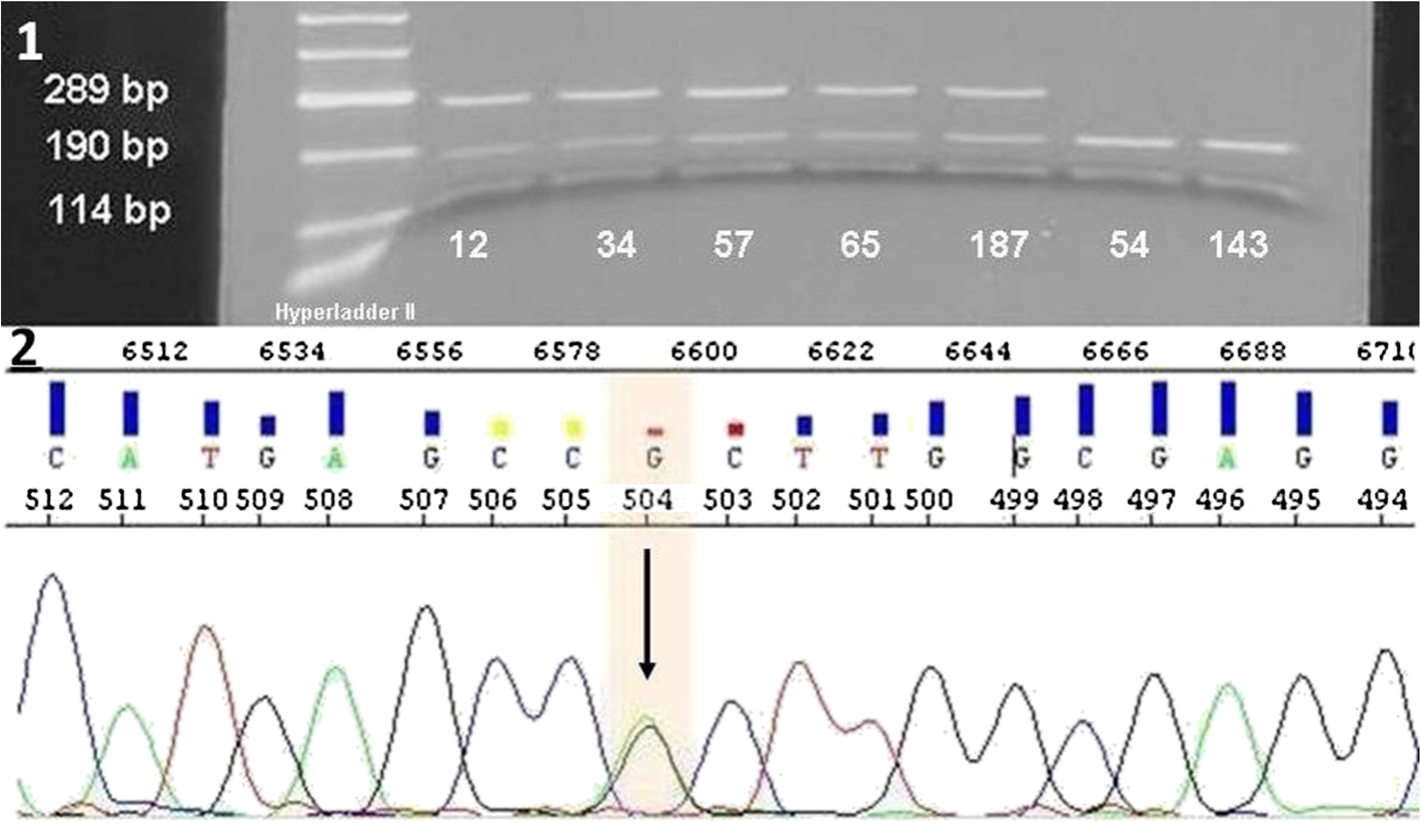
**Restriction analysis of *DNMT3A *R882H mutations. 1)** Agarose gel analysis of restricted products of 5 positive (12, 34, 57, 65, 187) and 2 negative (54, 143) patients. Wt samples showed 2 bands at 190 bp and 114 bp. Positive samples showed 3 bands at 289 bp, 190 bp, 114 bp because of the loss of a restriction site of Fnu4HI caused by the mutation. Hyperladder II (Bioline) was used as the marker. **2)** Representative sequence analysis of patient 187 showing heterozygote mutation CGC to CAC.

Figure 1 provides a representative result of restriction analysis with 5 positive and 2 negative samples. Point mutation at R882H (GCCGC to GCCAC) led to the loss of one recognition site of Fnu4HI, thus creating a larger 289 bp fragment. Because of heterozygosity, the 190 bp wt fragment and the smaller 114 bp fragment are present in every sample. Sensitivity of the assay was analysed using serial dilutions of wt and *DNMT3A* R882H-mutated DNA (initial mutation ratio in Sanger sequencing was 59%, Figure 2.1). The fragment containing the mutation was explicitly apparent with a mutational content of 0.05%, indicating a very high sensitivity of the assay. In addition mutations in exon 23 of *DNMT3A* were detected using HRM analysis. Results of HRM analysis were plotted as a difference in the fluorescence of the tested sample versus that of a wt control (normalisation line), referred to as a temperature-shifted difference plot (Figure 3.1). Discrepancies between mutated and wt samples could also be observed in the melting plot profiles. Sample containing R882H mutation showed 2 peaks at 84.5°C and 85.6°C, whereas the wt samples showed only 1 peak at 85.7°C. Compared to the wt allele, R882X allele was slightly shifted to the left, with a melting temperature of 85.6°C (Figure 3.2). Sensitivity of the HRM assay was assessed similar to that of restriction analysis. The assay had high confidence (97%-99%) for the mutated allele up to a mutation ratio of 5.9% (Figure 2.2). Lower mutation ratios could not be assigned as positive and were identified as false negative with a confidence of 92%-98%. Thus our results indicated that HRM analysis had a lower sensitivity than that of endonuclease restriction analysis but had the benefit of identifying different mutations in 1 PCR reaction.

**Figure 2:**
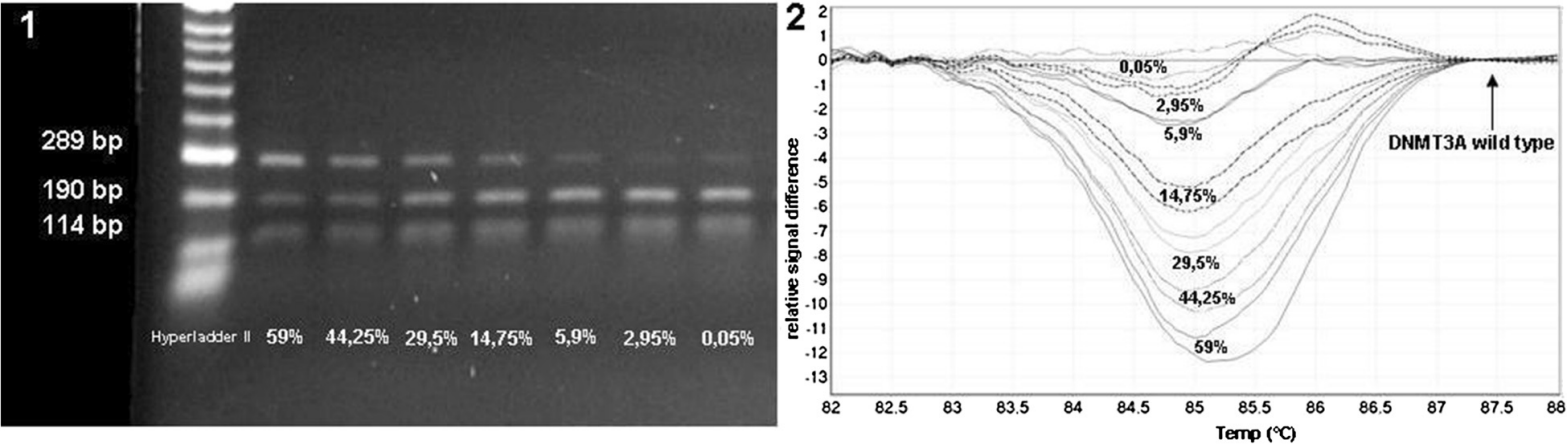
**Sensitivity analysis of *DNMT3A *R882H detection. 1)** Endonuclease restriction analysis of serial dilutions of *DNMT3A* R882H; Undiluted mutation ratio was 59% (estimated by sequencing). Mutated allele wa detected up to a degree of 0.05%. **2)** Difference plot for HRM analysis of serial dilutions of *DNMT3A* R882H: Correct estimation was possible up to a mutation ratio of 5.9%; lower mutation ratios were identified false-negative. Normalisation was performed to the wt allele.

**Figure 3:**
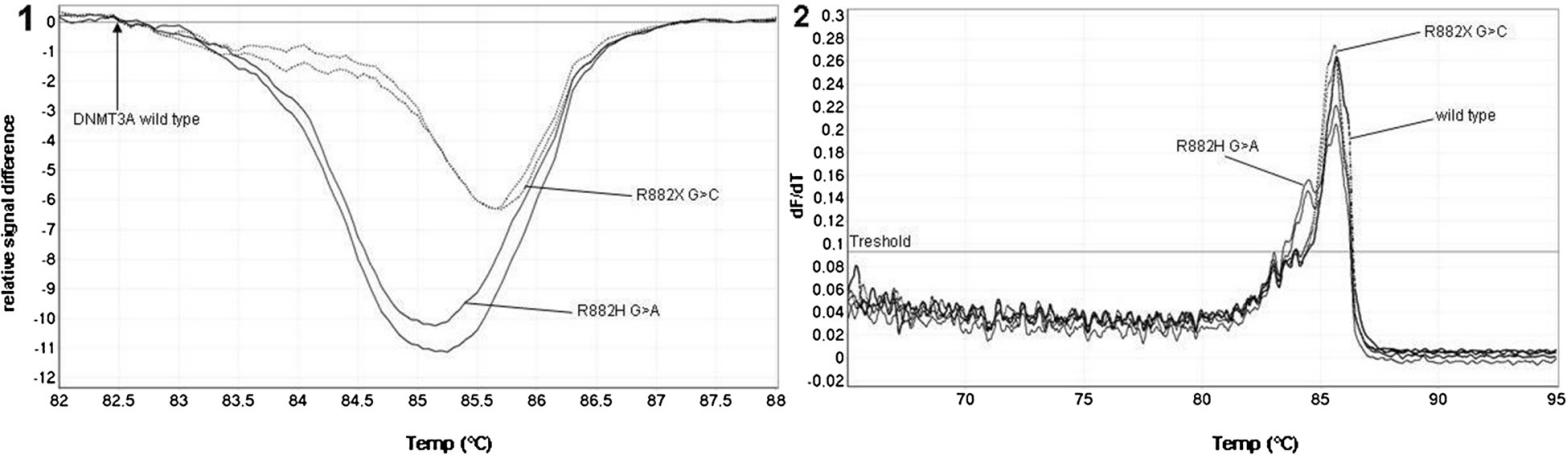
**HRM analysis of *DNMT3A *mutations. 1)** Difference plot for HRM analysis of *DNMT3A* R882H G>A and R882X G>C mutations. Normalisation was performed to the wt allele. R882X showed a right-shifted peak compared to R882H. **2)** Melting curve profiles of *DNMT3A* R882H G>A, R882X G>C and wt allele. Vertical axis corresponds to changes in the fluorescence signal over time (dF/dT). R882H G>A displayed 2 peaks (84.5°C and 85.6°C), while the wt allele had only one peak at 85.7°C. R882X G>C had a left shifted peak at 85.6°C.

### *IDH2* mutation analysis

The mutational frequency of *IDH2* R140Q G>A was 6.69% (16 out of 230 patients with AML), which was similar to the frequency published by Paschka et al. [23] and other studies [29,30]. Most patients with AML with *IDH2* mutations were older than 50 years and had *de novo* AML and a normal karyotype. Of 16 patients, 7 had an *NPM1* mutation.

The ARMS analysis allowed differentiation between mutated and wt DNA of *IDH2* through specific differences in the amplification properties of the reaction. In the presence of a mutation the PCR reaction generated 3 different fragments with sizes 613 bp (control band), 446 (mutation band) and 233 bp (wt band, Figure 4.1). No 446 bp mutation band was detected in the wt samples and results were confirmed by sequencing (Figure 4.2). In addition some faint unspecific bands of size ≥613 bp were detected. Given that the diagnostic approach was not handicapped, the assay was acceptable for further applications. HRM screening of *IDH2* showed no additional mutations in our AML patient group. *IDH2* amplification showed a bimodal melting profile with a smaller peak at 79.8°C and a bigger peak at 82.7°C. Differences in mutated and wt allele were visible during melting point analysis, because *IDH2* R140Q mutations shifted to lower temperatures than those in wt allele (Figure 5). Sensitivity tests were performed as those described for *DNMT3A*. For the *IDH2* allele, the sensitivities of ARMS and HRM analyses were the same, with a limitation at a mutational ratio of 2.25%. As shown in Figure 6.1 the mutation band at 446 bp was not present in dilutions with 2.25% and 0.45% mutated DNA. Furthermore, HRM analysis showed that dilutions from 45% to 4.5% were clearly positive with a confidence ranging from 77.68% to 98.41%, while the last 2 dilutions were false-negative, with a confidence of 82% to 94.39% (Figure 6.2).

**Figure 4:**
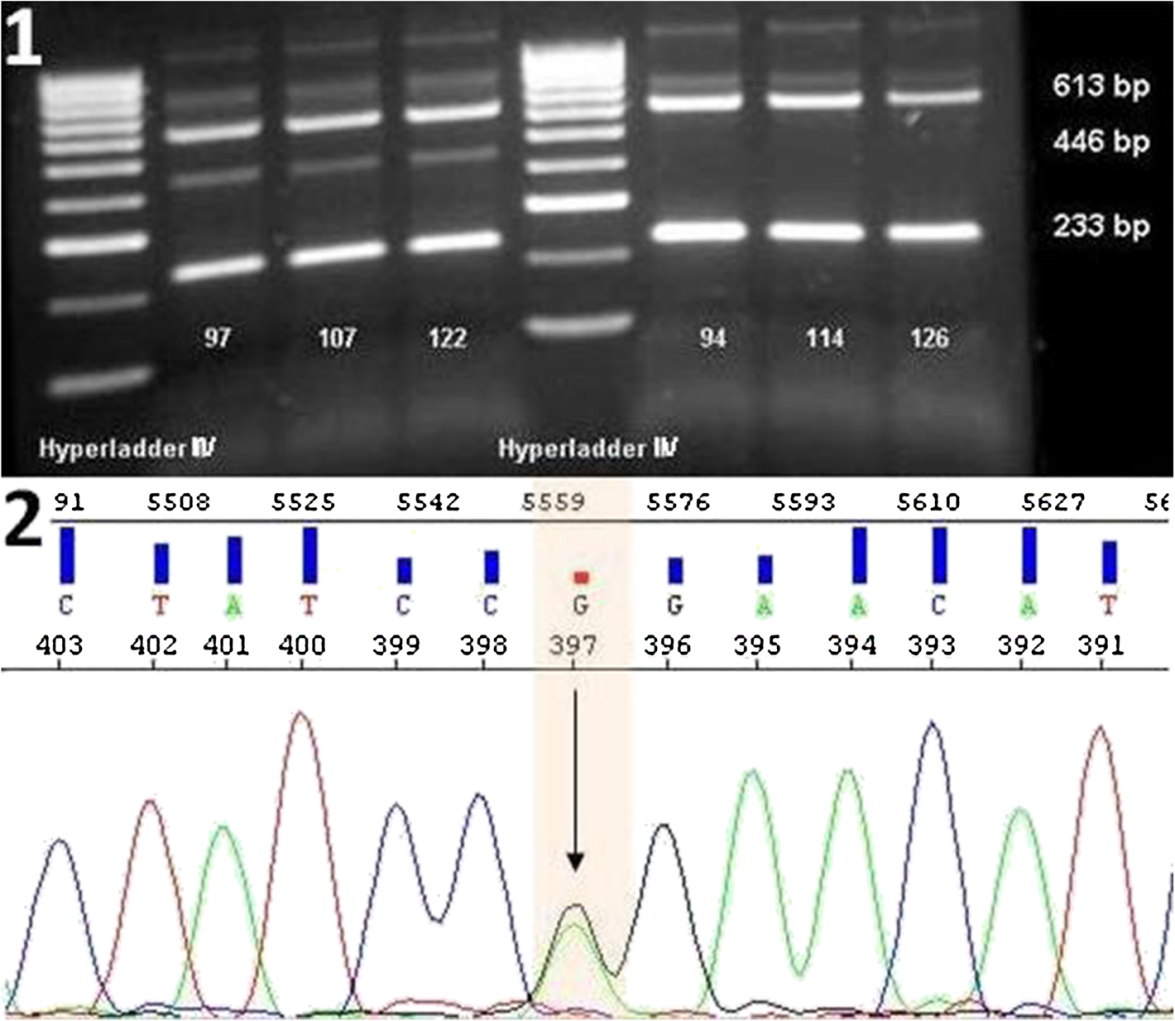
**ARMS analysis of *IDH2 *R140Q mutation. 1)** Agarose gel analysis of PCR products of 3 positive (97, 107, 122) and 3 negative (94, 114, 126) patients. All patients showed control (613 bp) and wt (233 bp) bands, while only the positive patients showed a product at 446 bp. Hyperladder II (Bioline) was used as the marker. **2)** Representative sequence analysis of patient 97 showing the heterozygote mutation CGG to CAG.

**Figure 5:**
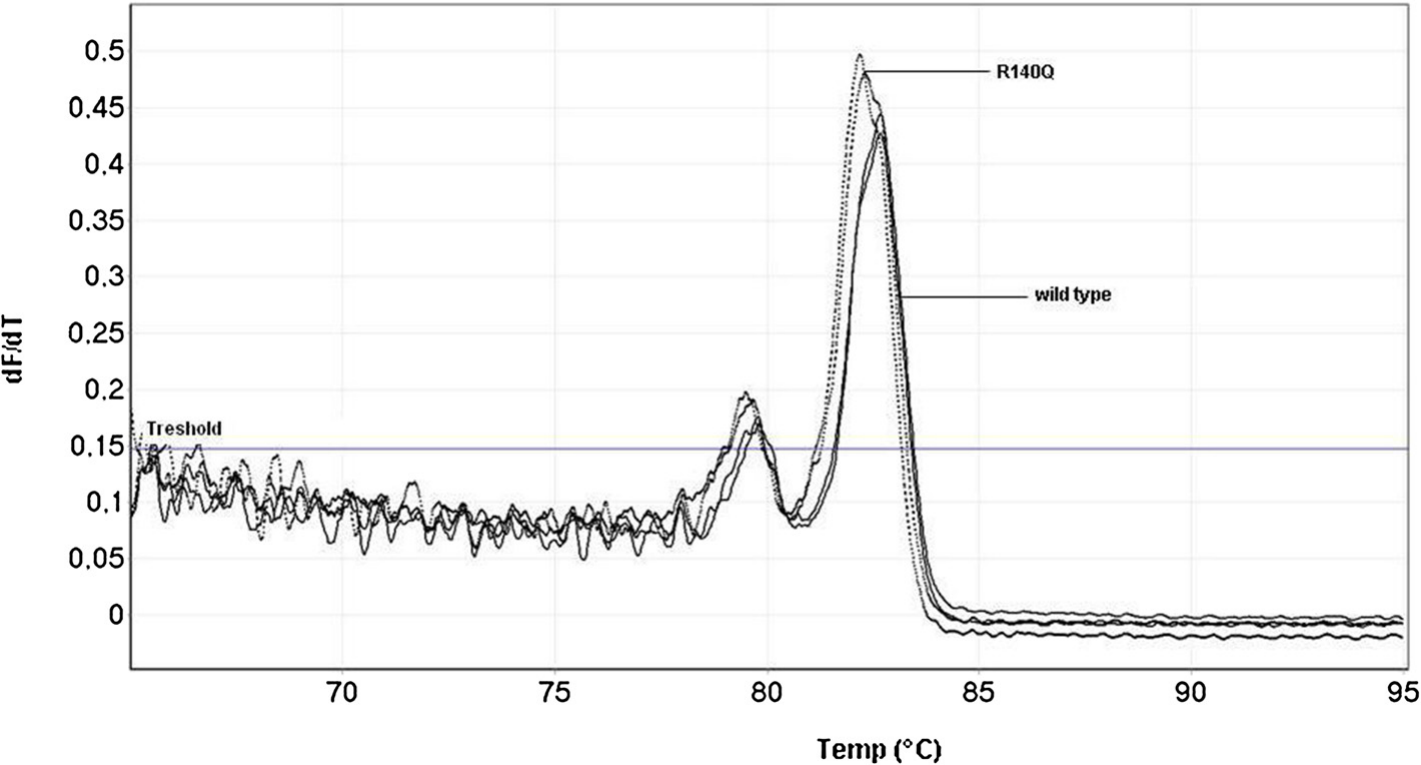
**Melting curve profiles of wt allele and *IDH2 *140Q G>A.** Vertical axis corresponds to changes in the fluorescence signal over time (dF/dT). *IDH2* analysis showed a bimodal peak; R140Q was shifted to lower temperatures compared to the wt allele.

**Figure 6:**
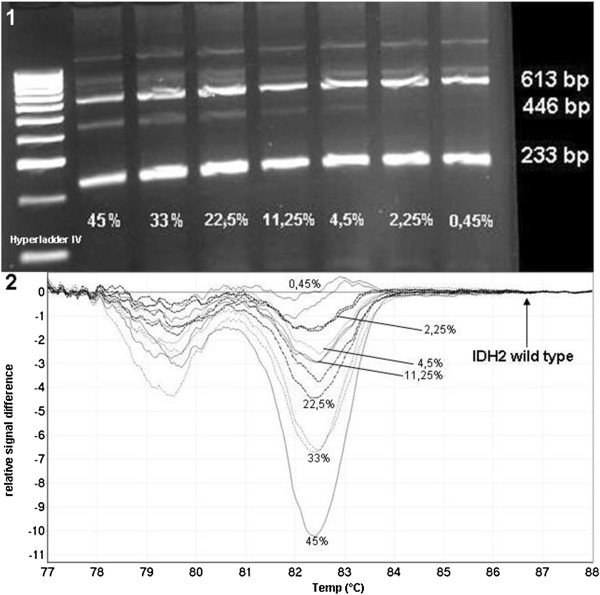
**Sensitivity analysis of *IDH2 *R140Q detection. 1)** Serial dilutions of *IDH2* R140Q: Undiluted mutation ratio was 45% (estimated by sequencing). Mutated allele was detected up to a degree of 4.5%. **2)** Difference plot for HRM analysis of serial dilutions of *IDH2* R140Q: Correct estimation was possible up to a mutation ratio of 4.5%; lower mutation ratios were identified false-negative. Normalisation was performed to the wt allele.

### *IDH1* mutation analysis

An assay to detect specific mutations is not applicable because of the heterogeneity of *IDH1* aberrations. Therefore, the HRM assay was evaluated for *IDH1,* as previously described by Patel et al. [30]. Mutated and wt *IDH1* was distinguished through their melting profiles because mutated DNA had a melting point between 80.3°C and 80.5°C while wt *IDH1* had a melting point of 81°C (Figure 7.1). However, the distinction between the different mutations of *IDH1* was difficult with this analysis as well as with the differentiation plot normalised to the wt control (Figure 7.2). During this study we observed that the temperature-shifted difference plot normalised to R132S C>A control sample was the best to determine different *IDH1* mutations (Figure 7.3). Thus, we performed sensitivity tests for G105 C>T and R132C C>T with normalisation to R132S C>A and for R132S C>A with normalisation to G105 C>T (Figure 8). HRM analysis showed sensitivity of 6%-7.8% for all three mutations. Using this method, we determined that 36 out of 230 (15.65%) patients with AML had *IDH1* mutations. Of these 19 (8.3%) had G105 C>T, 11 (4.8%) had R132C C>T and 6 (2.6%) had R132S C>A; this frequency is consistent with the data published by Nomdedéu et al. [22,29].

**Figure 7:**
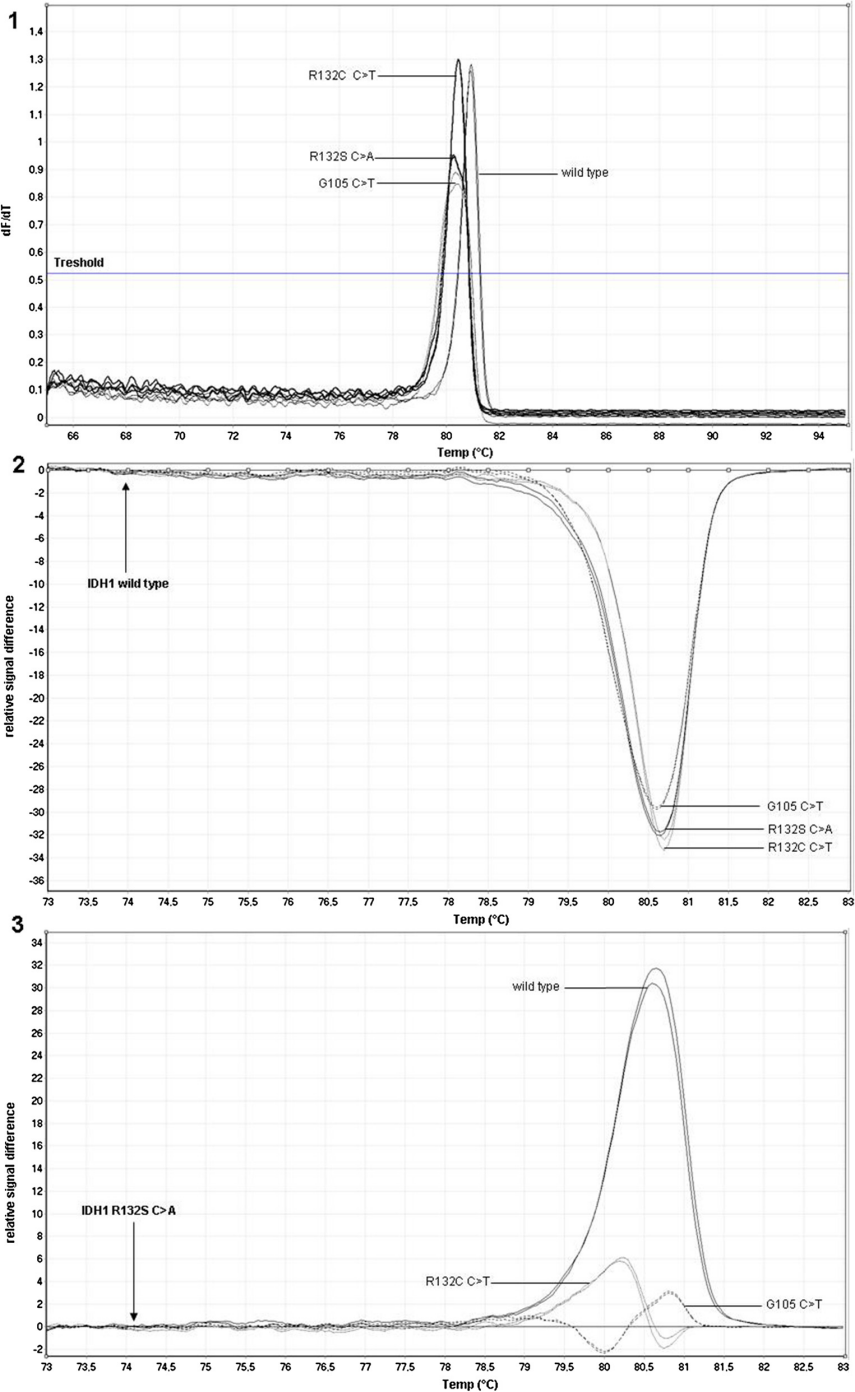
**HRM analysis of *IDH1 *mutations. 1)** Melting curve profiles of *IDH1* mutated and wt alleles. Vertical axis corresponds to changes in the fluorescence signal over time (dF/dT). Mutated alleles were shifted to lower temperatures, but differentiation between different mutations was not possible. **2)** Difference plot for HRM analysis of IDH1 mutations normalised to wt allele, discrimination of different mutations was difficult because of similar graphs. **3)** Difference plot for HRM analysis of *IDH1* mutations normalised to the R132S C>A allele, determination of different mutations was easier because of clearly separated graphs.

**Figure 8:**
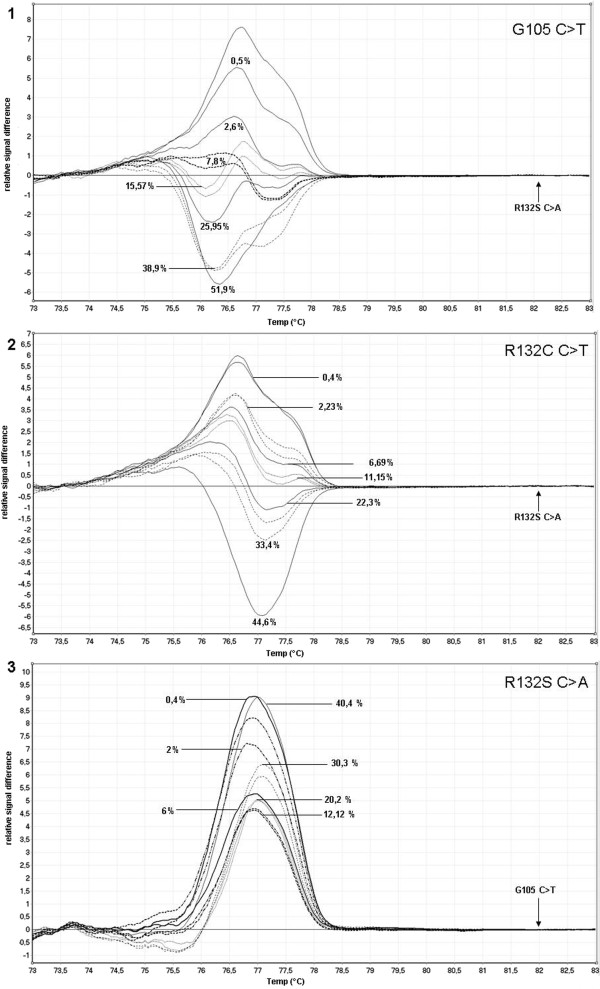
**Sensitivity analysis of different *IDH1 *mutations. 1)** Difference plot for HRM analysis of serial dilutions of *IDH1* G105 C>T: Undiluted mutation ratio was 51.9% (estimated by sequencing). Correct estimation was possible up to a mutation ratio of 7.8%; lower mutation ratios were identified false-negative. Normalisation was performed to the R132S C>A allele. **2)** Difference plot for HRM analysis of serial dilutions of *IDH1* R132C C>T: Undiluted mutation ratio was 44.6% (estimated by sequencing). Correct estimation was possible up to a mutation ratio of 6.69%; lower mutation ratios were identified false-negative. Normalisation was performed to the R132S C>A allele. **3)** Difference plot for HRM analysis of serial dilutions of *IDH1* R132S C>A: Undiluted mutation ratio was 40.4% (estimated by sequencing). Correct estimation was possible up to a mutation ratio of 6%, lower mutation ratios were identified false-negative. Normalisation was performed to the G105 C>T allele.

### Combination of different methods is essential to identify *DNMT3A* and *IDH1/2* mutations in routine laboratory analyses

Both the assays designed in this study for the detection of *DNMT3A* R882H and *IDH2* R140Q mutations were completely compliant with Sanger sequencing and had a high specificity. No false-positive results were determined with HRM analysis. Two (0.9%) samples showed variations for *DNMT3A* but were subsequently determined as wt by endonuclease restriction and sequencing. *IDH1* analysis with HRM showed that 6 (2.6%) samples had inaccuracies in melting profiles and hence were determined false negative with this method. Sequencing showed the presence of a R132C C>T mutation in this samples. *IDH2* analysis showed no discrepancies with Sanger sequencing.

Compared to Sanger sequencing, HRM analysis represents a timesaving, cost-efficient and more sensitive method to screen mutations in patients with AML at diagnosis. However, an efficient application presumes the presence of specific mutations and wt control samples. Because of the lack of cell lines with *DNMT3A, IDH2* and *IDH1* mutations, controls have to be established by sequencing different patient samples. Therefore, an effective application of HRM depends on the identification of high amounts of good-quality control samples, availability of a sequencer and HRM competent real-time PCR cycler. In addition, some results obtained with HRM analysis are difficult to interpret because of the variations in the melting curve of 1 mutation and can lead to uncertain conclusions or false-negative results [31]. Because new studies indicate the prognostic significance of *IDH1/2* and *DNMT3A* mutations, which affect the choice of therapy, a steady laboratory diagnosis is essential [10,17,18,21,22,32]. We developed ARMS-PCR to identify *IDH2* R140Q mutation and endonuclease restriction analysis to identify *DNMT3A* R882H mutations; both these methods are rapid and easy to use and interpret. Thus, these methods can be used to verify unclear results obtained using HRM analysis. In addition, these methods provide a possibility to identify the most common mutations in *DNMT3A* and *IDH2* in laboratories that do not have HRM-competent real-time PCR cyclers at their disposal. Secondary endonuclease restriction has higher sensitivity than HRM analysis that allows earlier identification of mutations at relapse during follow-up analysis [33]. For future applications this assay could also be adapted to the quantitative PCR (qPCR) technique. The forward primer can be modified to amplify only the genomic region containing the restriction position that is lost in the mutated state, thus allowing the exclusion of wt and mutated alleles as well as the quantitative assessment of *DNMT3A* mutation. The main characteristics of all the methods analysed in this study are summarised in Table 1.

The measured sensitivities depend on assay conditions and equipment. For example, small amounts of non-specific amplicons and different salt or inhibitory concentrations can influence assay sensitivity [34,35]. Therefore, each laboratory should validate the presented methods with their equipment before application. Both HRM analysis and ARMS-PCR had only low sensitivity, which possibly could lead to false-negative results. Therefore, low mutational ratios could be overlooked and these patients would receive an imprecise laboratory diagnostic report. Potential reduction of amplicon size for both HRM and ARMS analyses could optimise sensitivities [36]. Moreover, adaption of the qualitative endonuclease restriction assay to a quantitative assay could further increase sensitivity and provide objective measurements of mutated cells [37].

In the future, sensitivity limitations for screening *DNMT3A* and *IDH1/2* mutations can be overcome by using allele-specific next-generation sequencing (NGS). This method provides high multiplexing possibilities together with high sensitivity and broad spectrum of detected mutations [38]. However NGS is associated with high costs, high hands-on time and high computational expertise. Because standardisation and validation of NGS can be challenging establishment of this method is an ongoing process in laboratory routine [39]. Conventional PCR-based methods are easy to standardise and validate and therefore could be used when NGS is being implemented in order to provide routine mutational screening of patients with AML.

### Possible laboratory workflow for identifying *DNMT3A* and *IDH1/2* mutations

**Figure 9:**
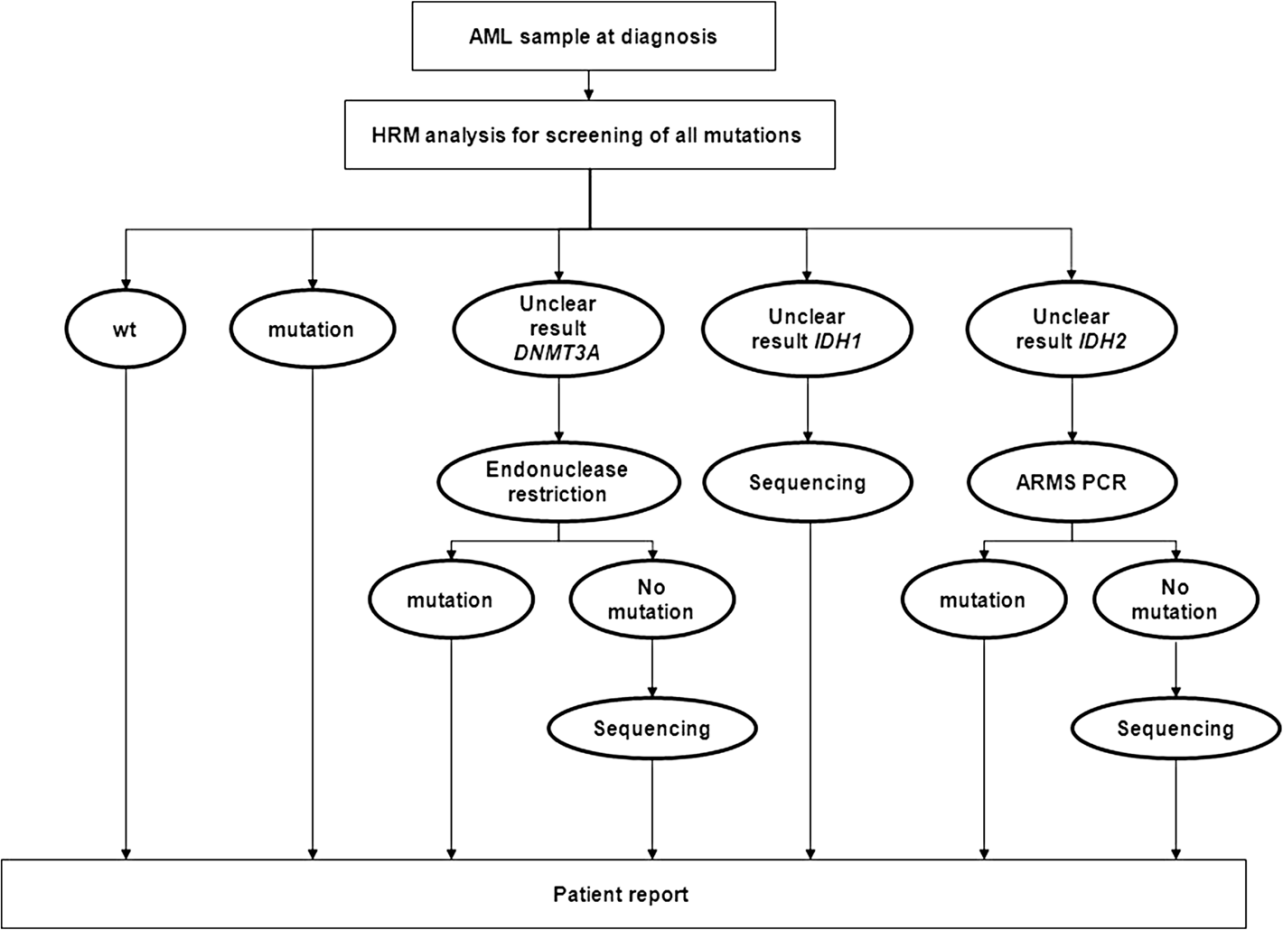
**Possible diagnostic workflow to identify *DNMT3A, IDH2 *and *IDH1 *mutations in routine laboratory analysis.** HRM analysis can be performed in the first diagnosis for all mutations because of high mutational ratios prior to therapy. Unclear results can be verified by endonuclease restriction or ARMS-PCR. Unclear *IDH1* results can be checked by sequencing because of the heterogeneity of possible mutations. Effective combination of all the available methods enables more reliable results and a cost-effective and time-saving routine laboratory analysis.

Although Sanger sequencing is considered as the “gold standard” for identifying mutations, this method is time-consuming and cost-intensive in routine laboratory practice, because the diagnostic findings need to be available as fast as possible. Based on the findings of this study, we developed a laboratory workflow for identifying *IDH1/2* and *DNMT3A* mutations in the first diagnosis and relapse without using of sequencing (Figure 9). HRM analysis should be the method of choice for differentiating between wt and all the analysed mutations in primary AML samples. In case of uncertainty results can be verified using the above presented methods. In addition, ARMS and endonuclease restriction provide a possibility to identify the most common *IDH2* and *DNMT3A* mutations when no HRM-compatible real-time PCR cycler is available. Because of the multiplicity of *IDH1* mutations, it was not possible to generate a valid method for analysing 1 specific mutation. For this reason HRM analysis is the best alternative to Sanger sequencing. After therapy, follow-up analysis should be chosen depending on the identified mutations at the first diagnosis. Because endonuclease restriction had higher sensitivity for R882H mutations, this method is more suitable for detecting low mutational ratio of known mutations in patients after therapy or relapse and progression of disease. Because of the ease of interpretation ARMS can also be used to identify *IDH2* R140Q mutations at relapse or disease progression.

**Table 1:** Comparative characteristics of all the methods used in this study

	***DNMT3A***			***IDH2***			***IDH1***	
	**Restriction endonuclease**	**HRM**	**Sanger sequencing**	**ARMS**	**HRM**	**Sanger sequencing**	**HRM**	**Sanger sequencing**
Sensitivity*, %	0.05	5.9	10	4.5	4.5	10	6 to 7.8	10
Turnaround time, days	1	1	2 to 3	1	1	2 to 3	1	2 to 3
Technician time, hours	4	3.5	10 to 12	3	3.5	10 to 12	3.5	10 to 12
Cost of diagnosis method, €	32.13	28	122	44.16	28	122	28	122
Interpretation	Easy	Medium -difficult	Medium	Easy	Medium -difficult	Medium	Medium -difficult	Medium
Identification of different/rare mutations	No	Yes	Yes	No	Yes	Yes	Yes	Yes
Special equipment	PCR cycler	HRM real time PCR cycler	Sequencer	PCR cycler	HRM real time real time PCR cycler	Sequencer	HRM real time real time PCR cycler	Sequencer

*Sensitivity was measured as the minimal percentage of mutated allele in a sample detected by the assay.

## Conclusion

In summary, we generated highly specific, sensitive and rapid methods for identifying the most common mutations in *IDH2* (R140Q) and *DNMT3A* (R882H), which can be used separately or in combination with HRM analysis to provide more reliable diagnostic results. All the developed methods were rapid, specific and easy to use and interpret. PCR-based methods are a useful tool for the routine laboratory identification of relevant prognostic mutations. We propose that early screening of mutations in patients with AML with normal karyotype could facilitate risk stratification and improve treatment opportunities.

## Abbreviations

AML: Acute myeloid leukemia; IDH: Isocitrate dehydrogenase; DNMT: DNA methyltransferase; HRM: High resolution melt; PCR: Polymerase chain reaction; α-KG: α-ketoglutarate; 2-HG: 2-hydroxyglutarate; NADP: Nicotinamide adenine dinucleotide phosphate; ARMS: Amplification-refractory mutation system; NPM: Nucleophosmin; FLT: fms-related tyrosine kinase.

## Competing interests

The authors declare that they have no competing interest.

## Authors’ contributions

BR carried out design of the study and drafted the manuscript. BO and BIW conceived of the study, and participated in its design and coordination and helped to draft the manuscript. KA and CR carried out the molecular genetic studies. SA and SC participated in sample collection and sequencing. All authors read and approved the final manuscript.

## Supplementary Material

Additional file 1: Table S1 Characteristics of patients with AML according to mutation status. Click here for file

Additional file 2: Table S2 Primers used in this study. Click here for file

Additional file 3 PCR reaction mixtures and conditions. Click here for file
