# Supplementary material for: Comparative examination of various PCR-based methods for DNMT3A and IDH1/2 mutations identification in acute myeloid leukemia
Source: J Exp Clin Cancer Res. 2014 May 21;33(1):44. doi: 10.1186/1756-9966-33-44 (PMC4045877; doi:10.1186/1756-9966-33-44)
Supplement: Additional file 1: Table S1 — Characteristics of patients with AML according to mutation status. [file 1756-9966-33-44-S1.docx]

**Table S1.** Characteristics of patients with AML according to mutation status

|  | **Total** | ***DNMT3A* mut+** | ***IDH1* mut+** | ***IDH2* mut+** |
| --- | --- | --- | --- | --- |
| **Characteristics** | 230 | 30 (13%) | 36 (16%) | 16 (7%) |
| Age (y), median (range) | 57 (16-94) | 58 (24-87) | 36 (19-85) | 63 (32-86) |
| M/F ratio | 116/114 | 11/19 | 18/18 | 9/7 |
| de novo AML, n (%) | 164 (71%) | 21 (70%) | 25 (69%) | 13 (81%) |
| Normal karyotype, n (%) | 115 (50%) | 16 (53%) | 20 (55%) | 10 (62%) |
| Favourable cytogenetics, n (%) | 18 (8%) | 0 | 3 (8%) | 0 |
| Adverse cytogenetics, n (%) | 64 (28%) | 9 (30%) | 7 (19%) | 3 (19%) |
| *NPM1* mutations, n (%) | 43 (19%) | 11 (37%) | 6 (17%) | 7 (44%) |
| *FLT3* mutations, n (%) | 66 (29%) | 16 (53%) | 6 (17%) | 2 (12%) |
| WBC count (10^9^x/L),  median (range) | 39.6 (0.4-400) | 46.9 (0.6-197) | 31.5 (0.5-182) | 39.9 (0.5-149) |
| BM blasts (%), median (range) | 72.7 (25-99) | 75.1 (25-95) | 71.5 (25-95) | 78.5 (30-95) |

*DNMT3A mut*+, presence of *DNMT3A* mutation; *IDH1 mut*+, presence of *IDH1* mutation; *IDH2 mut*+, presence of *IDH2* mutation; n indicates number of patients; AML, acute myeloid leukemia; WBC, white blood count; BM, bone marrow; Statistical analysis was performed using two-sided exact Fisher test. The cytogenetic risk groups were defined as follows: adverse risk, -5/5q-, -7/7q-, abn(3q) (excluding t(3;5)), t(11q23) (excluding t(9;11) and t(11;19)), abn(17p), complex aberrations (>4 independent aberrations); intermediate risk, patients without low risk or high risk constellations; favorable risk, t(15;17), inv(16)/t(16;16), and t(8;21) irrespective to additional cytogenetic abnormalities [[40](#_ENREF_40)].
