# Supplementary material for: Comparative examination of various PCR-based methods for DNMT3A and IDH1/2 mutations identification in acute myeloid leukemia
Source: J Exp Clin Cancer Res. 2014 May 21;33(1):44. doi: 10.1186/1756-9966-33-44 (PMC4045877; doi:10.1186/1756-9966-33-44)
Supplement: Additional file 2: Table S2 — Primers used in this study. [file 1756-9966-33-44-S2.docx]

**Table S2:** Primers used in this study

| **Name** | **Sequence** | **Application** | **Fragment size** |
| --- | --- | --- | --- |
| *DNTM3A*-Ex23F  *DNMT3A*-Ex23R | 5´-GTGTGGTTAGACGGCTTCC  5´-CTCTCCCACCTTTCCTCTG | Sequencing | 644 bp |
| *DNMT3A*-ResF *DNMT3A*-ResR | 5´-GTGATCTGAGTGCCGGGTTG  5´-TCTCTCCATCCTCATGTTCTTG | Restriction analysis | 444 bp |
| *DNTM3A*-hrmF  *DNTM3A*-hrmR | 5´- CCCTTACACACACGCAAAATAC  5´- CCCTCTCTGCCTTTTCTCC | HRM | 170 bp |
| *IDH1*-Ex4F  *IDH1*-Ex4R | 5´-GTTTAGGGTGTGCCAGTGC  5´-GTTGAGATGGACGCCTATTTG | Sequencing | 658 bp |
| *IDH1*-hrmF  *IDH1*-hrmR | 5´- GTCAAATGTGCCACTATCACTC  5´- GCCAACATGACTTACTTGATCC | HRM | 197 bp |
| *IDH2*-Ex4F  *IDH2*-Ex4R | 5´-GCTTGGGGTTCAAATTCTGG  5´-GAAAGGAAAGCCACGAGACAG | Sequencing | 534 bp |
| *IDH2*-FO  *IDH2*-RO  *IDH2*-FI  *IDH2*-RI | 5´-AATTGGGAGACTCCAGAGCCCACACATTT  5´-AGAGAGAGATGAAGAGACAAGCTGGGAGA  5´-GAAGATGTGGAAAAGTCCCAATGGAACTATCGA  5´-AAGACAGTCCCCCCCAGGATGTTGC | ARMS | 613 bp  446 bp  233 bp |
| *IDH2*-hrmF  *IDH2*-hrmR | 5´- GCTTGGGGTTCAAATTCTGG  5´- CTCTCCACCCTGGCCTAC | HRM | 249 bp |

HRM, indicates high resolution melt; ARMS, indicates Amplification-refractory mutation system
