# Supplementary material for: Comparative examination of various PCR-based methods for DNMT3A and IDH1/2 mutations identification in acute myeloid leukemia
Source: J Exp Clin Cancer Res. 2014 May 21;33(1):44. doi: 10.1186/1756-9966-33-44 (PMC4045877; doi:10.1186/1756-9966-33-44)
Supplement: Additional file 3 — PCR reaction mixtures and conditions. [file 1756-9966-33-44-S3.docx]

**Supplemental Methods: PCR reaction mixtures and conditions**

**Table S1: PCR mix of the ARMS analysis**

| **Reagent** | **Volume [µl]** |
| --- | --- |
| Water | 29,25 |
| 10x PCR buffer | 5 |
| MgCl_2_ (25 mM) | 6 |
| dNTPs (10 mM) | 1.25 |
| *IDH2*-FO (control primer, 10 pmol) | 1 |
| *IDH2*-RO (control primer, 10 pmol) | 1 |
| *IDH2*-FI (allele-specific primer, 10 pmol) | 2 |
| *IDH2*-RI (allele-specific primer, 10 pmol) | 2 |
| Ampli Taq Gold (5U/µl) | 0.5 |
| Genomic DNA | 2 |

**Table S2: PCR conditions of the ARMS analysis**

| **Temperature [°C]** | **Time [s]** | **Cycles** |
| --- | --- | --- |
| 95 | 600 | 1 |
| 95 | 60 | 35 |
| 65 | 30 |  |
| 72 | 45 |  |
| 72 | 600 | 1 |

**Table S3: PCR conditions of the endonuclease restriction analysis**

| **Temperature [°C]** | **Time [s]** | **Cycles** |
| --- | --- | --- |
| 95 | 600 | 1 |
| 94 | 30 | 35 |
| 60 | 30 |  |
| 72 | 30 |  |
| 72 | 600 | 1 |

**Table S4: PCR mix of HRM analysis**

| **Reagent** | **Volume** |
| --- | --- |
| 2x HRM Master Mix (Qiagen) | 12.5 µl |
| Forward primer (10 pmol) | 0.75 µl |
| Reverse primer (10 pmol) | 0.75 µl |
| Water | x µl |
| Genomic DNA | 50 ng |
| **Final volume** | 25 µl |

**Table S5: PCR conditions of the HRM analysis**

| **Temperature [°C]** | **Time [s]** | **Cycles** |
| --- | --- | --- |
| 95 | 300 | 1 |
| 95 | 15 | 45 |
| 62 | 60 |  |
| 72 | 10 |  |
| **Melting** | | |
| 65-98 | increase 0.05°C/s |  |

**Table S6: PCR mix of the first PCR cycler for DNA sequencing**

| **Reagent** | **Volume [µl]** |
| --- | --- |
| 10x PCR Buffer II | 5 |
| MgCl_2_ (25 mM) | 6 |
| dNTPs (10 mM) | 1.25 |
| Forward primer (10 pmol) | 1 |
| Reverse primer (10 pmol) | 1 |
| Ampli Taq Gold (5U/µl) | 0.5 |
| Water | 34.25 |
| Genomic DNA | 1 |

**Table S7: PCR conditions of the first PCR cycler for DNA sequencing**

| **Temperature [°C]** | **Time [s]** | **Cycles** |
| --- | --- | --- |
| 95 | 300 | 1 |
| 94 | 30 | 40 |
| 58 | 30 |  |
| 72 | 60 |  |
| 72 | 600 | 1 |

**Table S6: PCR mix of the sequencing reaction**

| **Reagent** | **Volume [µl]** |
| --- | --- |
| 5x PCR Buffer | 1.7 |
| BigDye | 2 |
| Forward or reverse primer  (10 pmol) | 1 |
| Water | 8.3 |
| Purified PCR product | 2 |

**Table S7: PCR conditions of the sequencing reaction**

| **Temperature [°C]** | **Time [s]** | **Cycles** |
| --- | --- | --- |
| 96 | 120 | 1 |
| 94 | 60 | 40 |
| 58 | 60 |  |
| 60 | 180 |  |
